# Appreciation Professor Johan W. Mouton, 3 November 1956–9 July 2019

**DOI:** 10.1093/jac/dkz500

**Published:** 2019-12-12

**Authors:** J Peter Donnelly

**Affiliations:** Editor-in-Chief


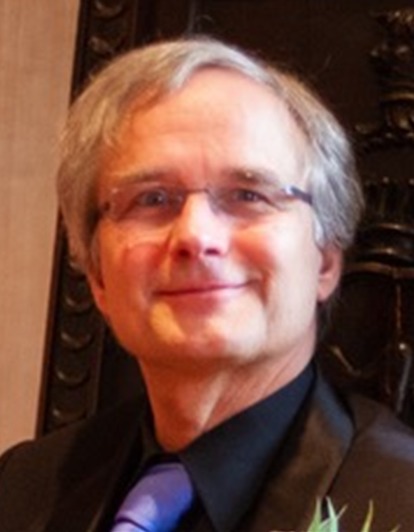


Johan Mouton has died aged 62 after a long illness. His death has clearly left a huge gap in the academic community as a whole, as has been attested by his obituaries in a range of media from a Dutch national newspaper, the *Volkskrant*, to various journals including *Lancet Infectious Diseases*, *Clinical Microbiology and Infection* and the *BMJ* as well as learned society websites such as ESCMID and the Dutch Society of Medical Microbiology (Nederlandse Vereniging voor Medische Microbiologie; NVMM). Each highlighted his immense energy, ebullient character, sense of fun, hobbies, and passion for and commitment to microbiology, whether in the laboratory, in research, teaching or when providing patient care. That he was devoted to his work was well known. He was a giant in the field of antimicrobial chemotherapy and, from first appearing on the global stage, he set out to expose microbiologists and others to his passions: pharmacokinetics and pharmacodynamics. An ardent member of the EUCAST team from 2001 onwards, he succeeded in incorporating these two disciplines into its definitions of antimicrobial susceptibility. His first successful encounter with *JAC* was when he published one of the papers that formed part of his PhD thesis (which was entitled *Pharmacokinetic and pharmacodynamic studies of β-lactam antibiotics in volunteers and patients with cystic fibrosis*) on one of the then-new carbapenems, meropenem.^1^ This early promise was more than fulfilled and heralded a very successful career, as evidenced by his manifold publications, contributions to textbooks, teaching activities, research and active participation in consensus groups.

Very soon he was offering his services as a reviewer for this and many other journals; something he regarded as a privilege and duty of every self-effacing scientist to help younger colleagues get their work published. A strong advocate for randomized clinical trials he also fully appreciated the role of animal models of infection in exploring pharmacodynamics to complete the triangle of patient–pathogen–drug in order to better understand how to obtain the best possible outcome. He was also passionate about antimicrobial stewardship and saw the value in producing practical treatment guidelines.

He joined the Editorial Board of *JAC* in 2015 and proved himself as industrious here as in all his other commitments. With his untimely death, the Journal has lost one of its most reliable Editors and experts; his shoes will prove hard to fill. On behalf of the Journal and its Editorial staff, I would like to extend our condolences and sympathy to his wife, Anouk, son, parents and siblings and all those who have lost a valued friend and colleague.
